# Community perceptions on the role of sexual activity on stroke: a qualitative study exploring the views of Ghanaian local community residents

**DOI:** 10.1186/s12889-019-7093-6

**Published:** 2019-06-10

**Authors:** Olutobi Adekunle Sanuade

**Affiliations:** Institute of Advanced Studies, University College London, Gower Street, WC1E6BT, London, UK

**Keywords:** Sexuality, Stroke, Sexual activity, Community, Ghana

## Abstract

**Background:**

Stroke is a major cause of morbidity and mortality in Ghana and sometimes comes with multifaceted complications including sexual dysfunction. While evidence is clear that living with stroke can result in sexual dysfunction, there are contradictory views regarding the causal association between sexual activity and stroke. This study explores perceptions of Ghanaian local communities on the role of sexual activity on stroke causation.

**Methods:**

This was a cross-sectional qualitative study. Thirty (30) focus group discussions (FGDs) were conducted in five communities across Ghana (Ga Mashie, Tafo, Gyegyeano, Chanshegu and Agorve) between October and November 2017. Data were analysed through a thematic approach.

**Results:**

Participants generally believed that sexual activity can cause a stroke. They mentioned that the dynamics through which sex can trigger a stroke include sex positions (i.e. having sex while standing and on the floor), high frequency of sex, having sex when older and engaging in indiscriminate sex.

**Conclusion:**

This study shows the need to pay critical attention to these community perceptions when developing intervention strategies for stroke in Ghana. This study also highlights that discussion about sexual activity in Ghana is more complex than the current health education programme allows, and so demands a ‘comprehensive sex education approach’ rather than a ‘disease-centered approach’.

## Background

The World Health Organization defined stroke as ‘rapidly developed clinical signs of focal (or global) disturbance of cerebral function, lasting more than 24 hours or leading to death, with no apparent cause other than of vascular origin’ [1]. Research shows that people who develop an acute stroke are at risk of developing a wide range of other complications, either as a direct result of injury to the brain, post-stroke disabilities, or stroke related treatments [2]. These complications may include brain edema, pneumonia, dysphagia, urinary tract infection (UTI), seizures, depression, bedsores, limb contractures, shoulder pain, and deep venous thrombosis (DVT) [2], and they may have significant impact on stroke prognosis.

Apart from the fact that stroke affects the physical bodies and the life trajectories of the sufferers [3], it can also affect the sexual function and satisfaction of both male and female sufferers. Stroke particularly impacts sexual function including libido, coital frequency, erectile and orgastic potency, and vaginal lubrication and sexual satisfaction [4]. Studies have shown that sexual activity after stroke may be affected by post-stroke depression, physical disabilities, inability to discuss sexuality with partners, general attitude towards sexuality, unwillingness to participate in sexual activity, side effects of medication, impotence or fear of impotence, concern over appearance, and emotional changes [4, 5].

Even though the various ways through which stroke complications can impact the sexuality of the sufferers have been extensively discussed in the literature, there is limited and inconclusive empirical evidence on how sexual activity can trigger a stroke among people with no history of stroke. While some studies discussed the mechanisms through which sexual activity might trigger a stroke, some argued that sexual activity may enhance cardiovascular health, and others showed no relationship between sexual activity and stroke onset. For instance, evidence showed that male sexual activity may trigger a haemorrhagic stroke in men because of increased heart rate, blood pressure and plasma nonadrenaline levels during sexual activity [6, 7]. Conversely, Ebrahim et al. (2012) showed that there is no association between frequency of sexual activity and incidence of ischemic stroke. The reason given for this is that during sexual intercourse, there is a decrease of cerebral blood flow in all cortical areas except in the right prefrontal cortex, and these changes are unlikely to precipitate an ischaemic stroke [8, 9]. Further, there is a body of evidence in Europe and North American showing that sexual activity enhances cardiovascular health and this is because sexual activity has a physical activity component which favours cardiovascular health [6, 10–13]. Hall and colleagues also argued the existence of indirect relationship between sexual activity and cardiovascular health because men who engage in frequent sexual activity are more likely to be in healthier relationships with a regular partner and this might improve cardiovascular health through stress reduction and social support [14].

Among the medical community, there is a stance that the underlying causal factors of stroke in patients that have experienced ‘sex triggered stroke’ include vasospasm [15], rupture of cerebral aneurysm causing cerebral haemorrhage or subarachnoid haemorrhage [16], and the risk factors include use of birth control pills, presence of foramen ovale [17] and undetected high blood pressure [18]. Therefore, it is clear from the medical standpoint that there is no concrete evidence that suggest a causal association between sexual intercourse and stroke. But the question is that how does local community residents perceive the role of sexual activity on stroke onset? This question is important because even though local community perceptions are often different from medical construction [19], they are important for developing primary prevention initiatives, as well as for stroke management and control [20]. Sociologists and anthropologists have particularly shown that every culture has its own explanations for causes of illnesses, and these often contradict the medical perception. While medical profession has the perception that disease is purely a biological condition, sociological and anthropological accounts revealed that diseases have social meanings [19, 21, 22].

Globally, no empirical research has explored cultural perception on how sexual activity could trigger a stroke. Nevertheless, there has been a lot of media attention and interest in Africa [23], Asia [24], Europe [25], and North America [26] on the relationships between sexual activity and stroke. Beliefs on sexual activity as a cause of stroke has only been mentioned in a study which explored perceptions on sexual pleasure and the construction of masculinities and femininities in Ghana [27]. Fiaveh and colleague showed that some urban residents in Ghana mentioned that sexual positions could trigger a stroke [27]; however, there was no detailed information regarding the mechanisms through which this could occur because the focus of their study was not to explore this association. This study therefore presents the perceptions of Ghanaian local community residents on the role of sexual activity on stroke causation. The information from this study is important because it may help scientific epidemiology derive hypotheses for studies that can make a strong contribution to public health programmes in Ghana. Evidence in Ghana showed that knowledge of local community perceptions of an illness provides important information for primary prevention strategies [28]. Also, the findings from this study elicit the importance of socio-cultural context in illness explanations.

### Context of sexual activity in Ghana

Since the medical notion of an illness is usually monolithic and sometimes contradicts local community perceptions [20], it is important to understand the context of sexual activity within the broader Ghanaian society. In Ghana, issues about sexual activity are looked at from the angle of morality [29] and subsumed under religion and social norms and values. Particularly, religion is at the centre of all discussions related to sexual behaviour, and the link between religion and morality is more or less inseparable. For instance, all the major three religions in Ghana (Christianity, Islam and Traditional) forbid premarital and extramarital sex and believe that there are sanctions for anyone who goes against this norm. From the religious perspective, discussion about sex in the presence of the youths is seen as encouraging immorality and the belief is that it can make the youths indulge in sex experimentation. As a result, churches often provide moral education instead of sex education [29]. While some researchers said that religious leaders constitute barriers to successful reproductive health in the Ghana, others argued that they are an important point of entry for successful interventions. Despite these arguments, there is a general consensus that religion matters when it comes to the issue of reproductive health and sexual behaviour in Ghana [30].

In the traditional Ghanaian society, sex discussion with youths was seen as a taboo and no formal sex education occurred in transition from youth to adulthood. Oppong and colleagues mentioned that any youth who tried to ask questions about sexual issues was seen as disrespectful and disobedient and would be insulted or ignored because these issues were for the adults only [31]. The traditional approach to sex education in Ghana included different puberty rites and these ceremonies basically marked the entry of young men and women into adulthood [29]. Those who went through these puberty rites were seen as decent and praised; however, girls with premarital pregnancies were seen as impure and looked down upon in the society. These forms of sex education existed to prevent promiscuity and premarital sex among youths and this was because many of the Ghanaian ethnic groups attached great importance to chastity [29]. In some of the traditional practices, while there were punishments for young women who engaged in premarital sex, men were treated very leniently. In most patrilineal societies in Ghana, marriage, through paying of the bridewealth, gives the man the exclusive sexual rights over the woman and it is culturally wrong for a woman to refuse sexual relations with her husband in such marriages [32]. This refusal could lead to divorce or gives permission to the man to engage in extramarital affairs.

Nevertheless, religious belief and traditional social control on sexual activities in Ghana now co-exist with the influence of education, urbanization, internet, and western media [33–35] thus leading to complexities of information received on sexual behaviour. Evidence showed that sexual communication among adults and adolescents is infrequent, however, the discussions that do occur are laced with threats and warnings or centre on key topics, primarily abstinence and HIV/AIDS [36, 37]. Currently, there is a growing sex consciousness and a perceived notion that Ghanaian society is becoming more sexualised than ever before. First, Ghana Demographic and Health Survey (GDHS) reports indicate a widened gap between median ages at first sex and first marriage across the past decade from 1.2 years to 2.3 years for women and 4.5 years to 6.4 years for men in 2003 and 2014, respectively [38, 39]. This suggests more engagement in premarital sexual activity than before. Second, sexually explicit images are projected and subtly woven into many areas such as adverts, billboards, media, local Ghallywood movies, other entertainment industries such as music and videos, and even when promoting regular products such as jeans, T-shirts, television, fragrance, etc. This growing sex consciousness triggered by sexually explicit images creates an avenue where sex is linked with almost anything including illnesses; a situation that did not exist in the country until about two decades ago.

Generally, traditional discourse in Ghana portrays men as sex-loving creatures. A study conducted among 12 to 19 year old adolescents in a poor urban community in Ghana found that although more girls reported being sexually active at those young ages, boys were more likely than girls to state their readiness for sexual activity [40]. Despite this, the changes taking place in the country also give women more ‘power’ to express and enjoy sexual pleasure just as men do. Fiaveh (2014) showed that the belief that women do not have the right to make sexual choices is not true as modern Ghanaian women have a high sexual agency [41]. Particularly, younger men and women who are married or are in stable relationships are willing to experiment with different sex positions and they learn more from local or international media activities. However, older men and women are conservative about this based on moral beliefs. Some Ghanaian women also have reservations for some sex positions. For instance, the missionary position (i.e. ‘man on top’ position) is perceived as the most appropriate during sex while anal sex is seen as sexual depravity. Fiaveh and Okyerefo (2017) showed that some community residents in Madina (an urban community in Accra) considered anal sex as bad sexual practice on grounds of morality, reproductive consequences (infertility), and stroke. These participants had the belief that sex positions such as ‘woman on top’ can cause harm to the reproductive organs, most especially to the uterus of a woman and to the male testicles and as a result affect the ability to conceive. A few of them also believed that standing sexual positions are a risky sexual practice which could lead to an illness such as stroke.

The question is- what is the foundation of the growing sex consciousness in Ghana? Since the emergence of HIV/AIDS in Ghana in 1986, much emphasis has been placed on the context within which sexual intercourse takes place in order to minimise HIV transmission. This is because more than 80% of HIV infections in the country are transmitted through heterosexual contact via unprotected sexual intercourse with infected person and non-use of condoms with multiple sexual partners [42]. As a result, HIV prevention messages and efforts in the country have focused on the following: promoting abstinence and faithfulness; promoting reductions in the number of sexual partners; encouraging delays in the onset of sexual activity among adolescents; promoting the correct use and consistent availability of condoms; strengthening programmes for STD control; and encouraging voluntary counselling and testing [38]. Perhaps, several discussions and extensive community interventions on sexual activity as a ‘trigger’ and ‘preventer’ of HIV/AIDs and other sexually transmitted infections (STIs) provide a basis where community residents started viewing sexual activity as a trigger of stroke. Or, is it possible that sexual activity is indeed a trigger of stroke after accounting for potential confounders? The aim of this study is not to answer this question but to show how community residents in Ghana are thinking about the association between sexual activity and stroke.

## Methods

### Design and setting

This was a cross-sectional qualitative study conducted in five regions in Ghana. While conducting a study on community perceptions on chronic disease and stroke in Ghana, the local residents mentioned factors such as hypertension, diabetes, poor lifestyles (e.g. excessive alcohol consumption, physical inactivity, and smoking), poor dietary practices, and supernatural factors (e.g. witchcraft, sorcery, curses, etc.) as causes of stroke and other chronic non-communicable diseases (NCDs). One thing that was striking during the group discussions was that many participants mentioned sexual activity as a major cause of stroke in Ghana. When this came up in the first community, this was further explored in the remaining four communities so as to have a comprehensive overview of community perceptions on the relationship between sexuality and stroke.

Data collection took place between October and November 2017. The aim of the broader study was to explore local community perceptions on chronic illness and stroke. Particularly, the study explored the following key areas: general life history; knowledge on health and illness; knowledge on definition, causes, management and prevention of chronic illness and stroke, and; social history of chronic illness. This paper therefore focused on local community perceptions on the different mechanisms through which sexual intercourse can trigger a stroke. Participants were mainly asked to narrate how sexual activity was related to stroke.

Participants were recruited through a purposive sampling process from five communities across Ghana. These include: Ga Mashie (Greater Accra region) Tafo (Ashanti region), Gyegyeano (Central region), Chanshegu (Northern region), and Agorve (Volta region). The five communities were selected to capture variations in stroke perceptions from major ethnic groups located in different geographical locations in Ghana. Ga Mashie, Gyegyeano and Agorve were located in the southern part of the country; Tafo was located in the middle-belt and Chanshegu in the northern part. In most of these communities, household living arrangements were poor and many youths lived in single parent household. These communities were characterised by poor economic circumstances, low education, and low access to healthcare. Many of the people in these communities were farmers and petty traders and characterised by low income earnings. The author had an established contact with Ga Mashie residents because of previous engagements with the community. As a result, it was relatively easy to recruit study participants in this community compared to others. In the remaining four communities, the author approached key ‘gatekeepers’ in each of these communities who helped in the recruitment of the study participants. These gatekeepers were members of the communities and had been involved in several data collection in their respective communities. As a result, they were able to help in recruiting participants that could provide detailed information regarding the focus of this study. The first step in the selection process was to travel around the communities in order to recruit people within similar age bracket and gender. After this, convenient venues (with easy access, and low distractions) were organised for the group discussions, and participants were subsequently informed. The initial plan was to have 10 people in each of the 30 groups. Hence, 300 people were expected to participate in the discussions; however, 45 people refused to participate. A total of 255 people participated in the study.

### Data collection

Data was gathered through Focus Group Discussions (FGDs) with local communities. Six (6) FGDs were conducted in each of these study sites, giving a total of 30 FGDs in the five communities and they were segmented by age and sex [20]. The FGDs were conducted in Ga (Ga Mashie), Twi (Tafo), Fante (Gyegyeano), Dagbani (Chanshegu) and Ewe (Agorve). All the group discussions were conducted by thirteen trained field assistants, with an average of two field assistants in each of the communities. The author was present in all the group discussions. The duration of the group discussions ranged from 90 to 150 min and permission was sought from the participants to record all the group discussions. All the participants were remunerated for participating in the study. The remunerations were provided after the data collection was completed. Hence, recruitment of participants for the study was based on voluntary participation. Details of the study areas have been provided in another study [20].

### Data analysis

The data were analysed through a thematic approach. All the group discussions were transcribed verbatim from Ga, Twi, Fante, Ewe and Dagbani into English language by a team of transcribers with Ga, Twi, Fante, Ewe, Dagbani and English language competence. All transcripts were coded using the ATLAS TI 7 and were analysed using thematic analysis. The first stage of the analysis involved reading all the transcripts to identify emerging codes and themes. A mix of deductive and inductive codes was employed for the analytical framework. The deductive codes were derived from previous studies that examined the relationship between sexual activity and cardiovascular events and studies on sexuality [8, 9, 27, 43, 44]. The author also had several discussions with a few colleagues who were experts in African history and sexuality. The reason for this was to ensure that the interpretations of the content of the codebook developed were objective and valid. The second stage of the analysis involved identifying the linkages between codes, themes, and appropriate respondent quotes, and existing research. First, four major themes on perceptions on the role of sexual intercourse on stroke were identified and these focused on frequency of sex, ageing, sex positions and indiscriminate sex (i.e. engaging in pre-marital or extra-marital sex). At the interpretive stage, the contents of each of these themes were examined to identify how they converge or diverge from previous medical and non-medical literature and to situate the participants’ responses within the broader Ghanaian context.

## Results

The findings focus on four thematic areas which emerged from the FGDs. These include: 1) frequency of sex and stroke; 2) sex positions and stroke; 3) ageing and stroke, and; 4) indiscriminate sex and stroke. The demographic characteristics of the participants are presented first. Variations in participants’ narratives by age and gender are presented in Table 1.

**Table 1:** Stroke perceptions by age and gender

Perceptions	Males (spread of views)	Females (spread of views)	Total (N)
Frequency of sex			
Youths (18–35 years)	Dominant (*n* = 32)	Dominant (*n* = 25)	57
Younger adults (36–59 years)	Dominant (*n* = 34)	Dominant (*n* = 21)	51
Elderly (60+)	Minority (n = 3)	Minority (n = 2)	5
Sex Positions			
Youths (18–35 years)	Dominant (*n* = 30)	Dominant (*n* = 43)	73
Younger adults (36–59 years)	Dominant (*n* = 28)	Dominant (n = 30)	58
Elderly (60+)	Minority (n = 2)	Minority (n = 6)	8
Indiscriminate sex			
Youths (18–35 years)	Absent (n = 0)	Absent (n = 0)	0
Younger adults (36–59 years)	Minority (n = 2)	Minority (*n* = 5)	7
Elderly (60+)	Minority (n = 4)	Minority (*n* = 8)	12
Old age sex			
Youths (18–35 years)	Absent (n = 0)	Absent (*n* = 0)	0
Young adults (36–59 years)	Absent (n = 0)	Absent (n = 0)	0
Elderly (60+)	Absent (n = 0)	Minority (*n* = 9)	9

### Demographic characteristics of participants

In total, 255 people participated in the FGDs and the number of participants in each of the FGDs ranged from 6 to 10. Participants’ age ranged from 18 to 75 years; the mean age was 43.4 years and 29.4% (*n* = 75) was below 30 years. The male/female ratio was 0.93:1. About one-quarter (*n* = 62) had never attended school, more than half (*n* = 153) had secondary education and only nine had tertiary education. Majority of the participants were involved in informal occupation such as trading and agriculture. Many of the participants (*n* = 124) reported that they were married. Most were Christians (*n* = 166) and the remaining were Muslims and Traditionalists. All the participants from Chanshegu were Muslims. Detailed profiles of the participants have been published elsewhere [20].

### Frequency of sex and stroke

When participants were asked to mention the causes of stroke, frequency of sex (i.e. too much sex) emerged in all the group discussions, except in Chanshegu. Participants mentioned that frequency of sex can cause stroke. Particularly, they explained that people who have sexual intercourse many times in a day or with concurrent partners are at higher risk of stroke. An adult woman in Ga Mashie said that she knew someone that was given several warnings by the doctor to stop concurrent sexual relationships; he ignored the warning and eventually developed stroke.

*Too much sex can also cause stroke…what he said is true. If you want to be having too many rounds of sex, about 10 in a day, it can make you get stroke (Young males, Agorve)*

*…still with the stroke, some people decide to have sex 4 or 5 times with their sexual partners in a day. This can lead to stroke (Young males, Gyegyeano)*

*I once asked someone, and the person told me that men who like engaging in sexual intercourse are the ones who get it (stroke) most. I don’t know why but that’s what I was told (Young females, Agorve)*

Some of the participants have the belief that God has endowed everyone differently in the sense that while some people have been given the ability to engage in regular sexual intercourse, some don’t have this ability. Hence, anyone who engages in too much sex without this ‘God-given ability’ can develop stroke in the process.

*It is too much of sex that gives stroke… one thing is that there is a difference in how God created everyone. Some people have the ability to have sex like 7 times in a day. Some can do it and there are others who do not have this ability. People who do not have this ability can develop stroke if they have sex many times in a day (Adult males, Ga Mashie)*

The narratives of the participants further showed that gender mediates the relationship between frequency of sex and stroke onset. They mentioned that men are more susceptible to stroke because they engage in too much sex. In addition, there was a community belief that during sexual intercourse, the woman gains ‘strength’ because she is a ‘receiver’ while the man loses strength because he is a ‘giver’; this is why men have higher risk of stroke. Women were seen to be naturally stronger than men when it comes to sex.

*Males have their causes of stroke and the females also have theirs. I know a sister in a town who got stroke because of her husband. She heard that her husband had married abroad. The shock of the news made her get stroke. But for us men, too much of sex causes our stroke (Elderly males, Tafo)*

### Sex positions and stroke

Regarding the relationship between sex positions and stroke onset, three types of sex positions were mentioned by the participants. These include: 1) having sex while standing; 2) having sex while kneeling, and; 3) having sex on the floor. Participants generally believed that sex positions, asides the “man-on-top position”, can result in stroke because they are not approved by God. Particularly, they mentioned that when a male and a female have sex while standing, this can result in stroke and the risk is higher for the man. They mentioned that the risk of stroke is higher for the man because with this sex position, he exerts a lot of energy as ‘the giver’, unlike the woman who is simply ‘the receiver’. Although this generated serious arguments during the discussions, there was a general consensus that having sex while standing can trigger a stroke.

Those who disagreed with this assertion were of the view that having sex while standing is a form of exercise and this should be a protective factor rather than a causal factor of stroke. Some also argued that if this is the case, then all the ‘White people’ should have developed stroke by now because they like having sex while standing. A few of the participants were genuinely not sure whether sex position is a cause of stroke even though they subscribed to this idea because it is a common knowledge in their communities; they were confused because they could not explain why women still have stroke since they are biologically stronger than men and do not exert a lot of energy during sexual activity.

*….when you are standing while having sex and the woman is standing too, it can cause stroke (Young males, Ga Mashie)*

While some of the participants mentioned that they have heard that doctors discourage having sex while standing, others said that they have seen people who developed stroke after taking this position during sex.

*You know, sometimes, you may meet a lady at night in the neighbourhood. Since people may pass around, you may not have enough time. So, you pin the lady against the wall and both of you stand to have sex. I have been told that doctors discourage this kind of sex position where both of you stand to have the sex (Young males, Tafo)*

*The way you have sex also counts a lot especially when you mostly do it in a standing position. I am saying this because it has happened to one of my brothers and up till now, he has stroke (Adult men, Agorve)*

Participants provided two mechanisms through which sexual activity while standing could result in stroke. The first reason is that when men have sex while standing, they exert a lot of energy and their ejaculations come with a force which predisposes them to stroke. Participants mentioned that this sex position weakens the veins around men’s waists and this invariably increases their risk of stroke. Secondly, some of the participants explained that men generally prefer sex positions such as “squat and penetrate” (i.e. kneeling position) and they believed that this sex position increases men’s risk of stroke. Some of the women in Tafo mentioned that their husbands dictate the sex positions they should be in and they don’t often refuse because if they do, the men will find other women willing to do so.

*Sex can cause stroke depending on the position of the man, precisely standing. When you ejaculate it comes with a force that can make you get stroke (Young males, Gyegyeano)*

*Yes. The reason is that some men stand to have sex with women. I hope you get me. You know when the man is ejaculating…..you know what I mean… I know you know what I mean.. As a young man continues to stand while having sex, he is not aware that he’s gradually nearing stroke in the future. He might say that’s the position he enjoys most but it’s leading to stroke (Young females, Gyegyeano)*

*That kind of sex position is a contributory factor. I hear there are some veins around their (men’s) waist, so, if they stand to have sex, it affects it and this can lead to stroke (Elderly females, Tafo)*

Further, participants in Ga Mashies mentioned that having sex while kneeling or having sex on the floor can result in stroke.

*…the kneeling is like you have bent your body whilst having sex. You see…, that is what brings about stroke (Elderly males, Ga Mashie)*

### Ageing, sexual activity and stroke

This theme came up only in Ga Mashie. Participants were of the opinion that there is an age limit to engaging in sexual activity. They mentioned that anyone, especially a man who continues to have sex having reached this age limit is at a higher risk of stroke. This narrative was particularly interesting because it came up only in the elderly female group discussions. The narratives of the elderly women groups focused on men as the gender that loves ‘sex’ and supposed to cease from engaging in sexual activity when they get to a particular age. There were several discussions on the fact that men like having sex even when they are old and they are the ones guilty of this. Some of the women went on to say that the elderly men in their community may even marry younger wives when they realise that their wives are either old or no longer attractive. They mentioned that one of the consequences of engaging in such behaviour is stroke and this is because their bodies are not strong (as before) to indulge in such sexual activity.

*Often times, sexual relationship between a man and a woman can result in stroke. This is because you will get to an age where you are not supposed to be in such relationships. If you do not listen to advice you can get stroke (Elderly females, Ga Mashie)*

*There is a certain age that when you get to, they will tell you the man that you are not supposed to have sexual relations with a woman. If you disagree and you go ahead to do it, it can give you stroke (Elderly females, Ga Mashie)*

### Indiscriminate sex and stroke

Indiscriminate sex was described as engaging in extramarital sex or being promiscuous. Ga Mashie participants believed that anyone who engages in indiscriminate sex (i.e. promiscuity) has a high risk of stroke. They mentioned that indiscriminate sex is a taboo in their community and indulging in such an act may either arouse the wrath of the gods or someone may cast a spell on another person for engaging in such act. They cited examples of some men who have been ‘given stroke’ because they slept with someone else’s wives and as a result, they were cursed by the real husbands of such women. The narratives of the participants also suggested that sometimes, indiscriminate sexual activity may not act alone to trigger a stroke but may act in conjunction with smoking and excessive alcohol consumption.

*…for instance, drinking, smoking, maybe at this my age I am living a promiscuous life, you see. You are being promiscuous, at the same time smoking and drinking. This can result in stroke (Elderly males, Ga Mashie)*

## Discussion

This study summarises the views of local Ghanaian community residents on the role of sexual activity on stroke onset. The narratives of the participants showed that the ways through which sex is believed to trigger a stroke include frequency of sexual activity, sex positions, having sex when older and engaging in indiscriminate sex. These findings are particularly interesting because they elicit how socio-cultural explanations of illness differ from medical explanations. This is because even though there is no medical evidence that indicates causal relationships between sexual activity and stroke, community residents held the belief that this cause-and-effect relationship exist.

What medical evidence has shown so far is that male sexual activity may trigger a haemorrhagic stroke (but not caused it) [18] and the fact that no association exist between sexual activity and ischemic stroke [8]. Some have argued that linking sexual activity with stroke onset is a misconception and this can arise from the fact that stroke mostly occurs in the morning while sexual activity mostly takes place at night and early in the morning [8]; hence, local community residents can easily make this causal link. While the purpose of this study is not to provide a scientific or medical basis for the association between sexual activity and stroke onset, it reinforces the importance of paying critical attention to socio-cultural explanations of illness when developing intervention strategies.

Participants mentioned that having sex while standing, kneeling, or on the floor can result in stroke. Some Ghanaians still hold the belief that the most appropriate sexual position within the context of marriage is the missionary position. However, other sex positions such as sex while standing, kneeling or on the floor are perceived to take place during sexual activity in ‘out-of-ordinary’ locations or in extramarital affairs. These ‘out-of-ordinary’ sex positions are seen as immoral and perceived to have disease consequences [27]. Consequently, male sexual activity in these positions may perhaps raise the heart rate and blood pressure which is likely to trigger a haemorrhagic stroke in susceptible individual.

It is interesting that when participants explained the role of indiscriminate sex on stroke, there was no discussion on premarital sex as a cause of stroke; the entire discussions centered on extramarital sexual activity. This may probably be due to the increasing rates at which premarital sex is becoming normalised in the Ghanaian society and so people’s views about premarital sex are more permissive. However, for married individuals, social norms dictate that the person is expected to be faithful to his or her partner. The society particularly has permissive stance towards a man having an affair with a single woman, but if the woman is married, then this becomes a problem. In Ghana, many religious denominations hold the belief that premarital and extramarital relationships are sinful and have consequences which may include being struck with an illness. There is also the traditional belief that any man who sleeps with another man’s wife or daughter may be struck with a thunderbolt or can be bewitched. Hence, some of the local community members believe that some people who have stroke may have been ‘stricken’ by the illness as a result of their promiscuous lifestyle. Also, medical literature showed that engaging in extramarital affairs can increase the risk of cardiovascular events [44] and some of the reasons for this include:1) extramarital sex usually occurs with someone younger than the primary partner and so may have negative effect on cardiovascular health since it often takes place after excessive drinking and/or eating [45]; 2) extramarital sex in an unfamiliar setting may significantly increase blood pressure and heart rate, leading to an increased myocardial oxygen demand (46]; 3) the enhanced physiological response to coitus during extramarital sex might trigger the fracture or erosion of a vulnerable pre-existing atheroma plaque, resulting in sudden death or non-fatal cardiovascular event [46, 47]; 4) the feeling of guilt that comes after extramarital sex or deceiving a sexually available and involved partner may induce psychological distress which may increase cardiovascular risk [46, 48]. These studies showed that both medical literature and local community perceptions suggest that engaging in extramarital affairs may increase the risk of cardiovascular disease such as stroke.

Further, participants mentioned that sexual activity during old age increases the risk of stroke. This may probably arise from the latent norm and perception that depict the elderly as asexual and weak, and the youths as strong and virile. According to Van der Geest (2001), sex in Ghana is regarded as a matter of ‘strength’ and meant for the youths only [49]. Some researchers have linked this ‘misconception’ to the activities of the media which portray elderly people as frail and lacking sexual feelings [50]. In the same way, there are increasing media advertisements on the use of sex enhancing products and many of these mostly target youths or young adults. This indicates that an elderly person who wants an active sexual life may be seen as depraved or lustful. This notion therefore makes it easier to see ‘old age’ sexual activity as socially unacceptable and anyone who engages in such activity may suffer a stroke as a consequence. Another plausible explanation for this finding is that since advancement in age is positively associated with higher prevalence of vascular risk factors [51] and age-related physical and psychological illnesses [50, 52], there is a possibility that these risk factors might be triggered during sexual activity of an elderly person thus leading to a stroke. This is not unlikely because empirical studies revealed that many older people engage in sexual activity even to their eighties and nineties [50].

The findings further showed that participants’ narratives regarding stroke perceptions varied by age and gender (Table 1). With respect to age, many of the youths and younger adults associated frequency of sex and sex positions with stroke compared to the elderly. While the younger adults and the elderly associated indiscriminate sex with stroke, only the elderly participants in Ga Mashie linked old age sexual activity with stroke. Regarding gender, while many of the male participants mentioned that frequency of sex can cause stroke, many of the females associated stroke with sex positions. Indiscriminate sex as a causal theory of stroke came up in many of the narratives of the female participants compared to the male participants. Furthermore, only the female participants associated old age sexual activity with stroke. Finally, the general perception among the participants was that men get stroke mainly because of sex and women because of other factors that may or may not directly relate to sex. It was also implicit in most of the discussions that men lose strength during sex and get stroke, while women gain strength. The repercussion of this perception is something that needs to be explored and understood.

### Implications of the findings

Studies have shown the importance of understanding local community perceptions of illness because this has implications for developing primary interventions and health promotion programs [20, 53]. The growing sex consciousness in Ghana, mainly through the activities of the media (both local and foreign), has created a mix of ideas on sexual activity regarding what, why, how, where, and when it should occur as well who to have sex with. This shows how complex issues on sexual activity have become in the country, with reducing effects of religion and traditional norms and increasing impact of Westernization and globalization. Also, lack of adequate sex education may be a plausible explanation for the participants perceptions on the role of sexual activity on stroke. This indicates that the current sexual health education structure which focuses exclusively on prevention of HIV/AIDS, other sexually transmitted infections, and unwanted pregnancies may not suffice in this growing era of sex consciousness. The perceptions of many of the participants in this study indicate that discussion about sexual activity in Ghana is more complex than the current health education programme, and so demand a ‘comprehensive sex education approach’ rather than a ‘disease-centered approach’. This study recommends the need to extend discussions about sexual activity in Ghana to other areas/ issues including noncommunicable diseases.

Further, it is possible that participants’ perceptions on the role of sexual activity on stroke were partly influenced by their inability to distinguish between real stroke and stroke mimics (non-vascular conditions that present with an acute neurological deficit simulating acute ischemic stroke) [54, 55]. Some of the most common stroke mimics include seizure, syncope, sepsis, migraine (including sex-linked headaches or orgasmic migraine), space-occupying lesions, functional disorders and metabolic conditions [56, 57]. Hence, it is possible that some of the sex-linked stroke cases described by the participants were stroke mimics. Therefore, it is important for further studies to explore community residents’ knowledge on stroke mimics and real stroke. Also, further studies need to be done to examine the prevalence of stroke mimics in Ghanaian hospitals: this is important to avoid unnecessary acute treatment and secondary prevention at the emergency department [54].

### Limitation and strength

The main limitation of this study is that the study used a convenience sample, and as a result, the findings from this study cannot be generalised to the whole of Ghana. Nevertheless, this study has provided comprehensive information on local community perceptions on the relationship between sexuality and stroke. This is an important social issue that needs to be addressed in a seemingly highly sexualised society like Ghana.

## Conclusion

This study provides interesting insights on the relationship between sexuality and stroke from local community perspectives. Findings showed that for primary intervention strategies on stroke to be successful in Ghana, socio-cultural explanations on causal theories of stroke need to be taken into consideration. Also, future empirical studies need to explore whether sexual activity might be a potential cause of stroke or tease out the mechanisms through which sexual activity might trigger a stroke. This kind of research will help to enlighten the research community as well as policymakers and will also help to see areas of convergence and divergence between medical construction and socio-cultural explanations of stroke.

## Data Availability

The datasets used and/or analysed for this study are not available on a public repository as they contain identifiable and sensitive information making it impossible to protect participants’ confidentiality. Researchers interested in accessing this data may contact the author.

## Abbreviations

AIDS: Acquired immune deficiency syndrome
DVT: Deep venous thrombosis
FGDs: Focus group discussions
GDHS: Ghana Demographic and Health Survey
GHS-ERC: Ghana Health Service Ethics Review Committee
HIV: Human immunodeficiency virus
NCDs: Non-communicable diseases
REC: Research Ethics Committee
STIs: Sexually transmitted infections
UTI: Urinary tract infection
